# Extracorporeal Shock Wave Therapy Combined with Subtalar Arthroereisis and Medial Column Stabilisation in the Treatment of PCFD: A Retrospective Study

**DOI:** 10.1007/s43465-026-01756-8

**Published:** 2026-03-12

**Authors:** Guanglong Zeng, Qingxiang Xie, Haiquan Mai, Liu Zhang, Boyuan Su

**Affiliations:** Department of Orthopedics, Dongguan Hospital of Traditional Chinese Medicine (Dongguan Hospital of Guangzhou University of Chinese Medicine), Dongguan, Guangdong China

**Keywords:** Progressive collapsing foot deformity (PCFD), Subtalar arthroereisis (HyProCure) and medial column stabilisation, Extracorporeal shock wave therapy (ESWT)

## Abstract

**Objective:**

Both the stability of the subtalar joint and the support of the medial column serve as crucial stabilising structures in progressive collapsing foot deformity (PCFD). Effective restoration of these stabilising components—often involving gastrocnemius or Achilles tendon recession—is essential for the treatment of PCFD. Therefore, this study aims to evaluate the clinical outcomes of extracorporeal shock wave therapy (ESWT) combined with subtalar arthroereisis and medial column stabilisation for PCFD.

**Methods:**

Sixty-eight patients (68 feet) with PCFD treated at our hospital between February 2023 and April 2024 were retrospectively analysed. The patients were divided into two groups based on treatment regimen: the intervention group (IG, *n* = 36), which received ESWT combined with subtalar arthroereisis (HyProCure) and medial column stabilisation [hereinafter referred to as ‘dual stabilisation procedures (DSP)’], and the control group (CG, *n* = 32), which underwent DSP alone. The groups underwent preoperative and postoperative assessments of clinical function and imaging parameters. Clinical function assessments included the Visual Analogue Scale (VAS) for pain, American Orthopaedic Foot and Ankle Society (AOFAS) ankle–hindfoot score, and Tegner activity scale score. Conversely, imaging assessments encompassed weight-bearing anteroposterior talo-first metatarsal angle (T1-MT), talonavicular coverage angle (TNCA), lateral calcaneal pitch angle (pitch angle), lateral talo-first metatarsal angle (Meary’s angle), calcaneal valgus angle (CVA), and elastic modulus of the Achilles tendon or gastrocnemius muscle. Complications, including flatfoot recurrence, internal fixation implant rejection, and wound infection, were also documented.

**Results:**

Postoperative follow-up lasted at least 12 months, with a mean duration of (13.66 ± 1.65) months. At the last follow-up, analyses revealed that both groups exhibited significant improvements in weight-bearing X-ray parameters (T1-MT, TNCA, pitch angle, Meary’s angle and CVA) compared with their preoperative values (*P* < 0.05). The intervention group demonstrated superior outcomes for CVA and elastic modulus of the Achilles tendon and gastrocnemius muscle compared to the control group (*P* < 0.05). Postoperatively, in both groups, the AOFAS and Tegner activity scores were higher than the preoperative values within the same group, whereas the VAS scores were lower relative to the preoperative measurements. Notably, the intervention group showed higher AOFAS and Tegner activity scores than the control group at 2 and 6 months postoperatively and at the last follow-up (*P* < 0.05) and a lower VAS score at 2 months postoperatively (*P* < 0.05). During follow-up, 59 patients (86.76%) from both groups met the criteria for returning to athletic activity. The intervention group achieved a shorter time to return to athletic activity [(6.26 ± 1.26) months; 33 cases, 91.67%] compared with the control group [(8.24 ± 2.06) months; 26 cases, 81.25%], with a significant difference (*P* < 0.05). Postoperatively, one patient in the intervention group developed recurrence of flatfoot deformity after removal of the subtalar joint stabiliser, and one patient in the control group had poor healing of an infected medial foot wound. No other severe adverse complications were observed in either group.

**Conclusion:**

ESWT combined with DSP effectively ameliorates PCFD, restores ankle–foot function and lower limb alignment and facilitates earlier return to athletic activity postoperatively.

## Introduction

Flexible flatfoot presents as a normal medial arch in the non-weight-bearing position but collapses under load, often causing medial foot pain, forefoot abduction, hindfoot valgus, subtalar instability and gait abnormalities [1]. If unmanaged, it may progress to posterior tibial tendon (PTT) degeneration, spring ligament laxity and other secondary deformities. Achilles or gastrocnemius tightness frequently coexists and accelerates deformity progression [2]. Conservative measures—including anti-inflammatory medication, rehabilitation, arch supports and orthopaedic footwear—may relieve symptoms, but failure can lead to worsening deformity and eventual transition to rigid flatfoot requiring surgical management [3].

Historically, flatfoot deformity has been attributed to posterior tibial tendon (PTT) insufficiency, which leads to progressive medial arch collapse and secondary deformities. In 2020, the term progressive collapsing foot deformity (PCFD) was introduced to more accurately represent its multi-structural and progressive nature. Subtalar arthroereisis with HyProCure restricts pathological subtalar motion, corrects hindfoot valgus, prevents talar lateral subluxation and reduces strain on the PTT, making it an important option for flexible flatfoot management [4]. Medial soft-tissue stabilisation, including PTT repair or reconstruction, spring ligament (SL) reinforcement and accessory navicular procedures, further restores medial arch support, with some cases requiring adjunctive bony procedures such as talonavicular fusion or Cotton osteotomy [5, 6].

Gastrocnemius or Achilles contracture frequently coexists in PCFD, increasing hindfoot valgus and contributing to arch collapse. Although surgical release may improve dorsiflexion, it carries risks such as sural nerve injury, postoperative weakness, adhesions and cosmetic concerns [7]. Extracorporeal shock wave therapy (ESWT) has recently gained attention for its ability to reduce myofascial contracture and improve tendon elasticity, showing therapeutic potential in flexible flatfoot management [8]. However, no study has evaluated ESWT in combination with HyProCure and medial column stabilisation (DSP). This study therefore aimed to assess the clinical efficacy of ESWT combined with DSP in patients with PCFD.

## Methods

### Study Participants and Grouping

The clinical data of 68 patients (all with single-foot involvement) diagnosed with PCFD complicated by Achilles tendon/gastrocnemius aponeurosis contracture admitted to our hospital between February 2023 and April 2024 were retrospectively analysed. The patients were stratified into two groups based on treatment regimens: the intervention group (*n* = 36) receiving ESWT combined with DSP and the control group (*n* = 32) undergoing DSP alone. This study was approved by the Ethics Committee of Dongguan Hospital of Guangzhou University of Chinese Medicine [Approval No. Dongguan TCM Ethics Review (Research) PJ [2023] no. 68]. The patients signed the consent form after being fully informed.

### Diagnostic Criteria

1. Criteria for flatfoot [9]: On weight-bearing lateral foot X-ray: talo-1st metatarsal angle (Meary’s angle), a normal foot is defined as 0° ≤ Meary’s angle < 4°, whereas flatfoot is defined as Meary’s angle ≥ 4°; and calcaneal pitch angle (pitch angle), a normal foot is defined as 20° ≤ pitch angle < 30°, and flatfoot is suspected as 10° ≤ pitch angle < 20°. Flatfoot is diagnosed when both criteria are met.
2. Criteria for flexible flatfoot [10]: The patient undergoes a heel-rise test in a weight-bearing position. Flexible flatfoot is diagnosed if the medial arch collapses during standing but is restored after heel elevation.
3. The 2020 AOFAS consensus on the new classification of progressive arch collapse deformity (PCFD) was adopted [11], and included patients who were classified as PCFD stage I (flexible), types A–D.
4. Positive findings on the Achilles tendon contracture test or SilfverskiÖld test [12]: Gastrocnemius contracture is indicated if the ankle dorsiflexion angle is < 10° with the knee extended or if the difference in ankle dorsiflexion angle between knee extension and flexion exceeds 13°; Achilles tendon contracture is suggested if the ankle dorsiflexion angle is < 10° irrespective of knee extension or flexion.

### Inclusion Criteria

(1) Meeting the diagnostic criteria for flexible flatfoot and PCFD staging, with complete medical records and capacity to comply with follow-up protocols; (2) aged 12–75 years; (3) preoperative magnetic resonance imaging confirming injury or degeneration of PTT or spring ligament; (4) positive findings on the Achilles tendon contracture test or SilfverskiÖld test; and (5) patients who completed follow-up.

### Exclusion Criteria

(1) Rigid flatfoot; (2) age outside the range of 12–75 years; (3) complicated with neurological diseases, lower limb sensory disturbance or hypofunction; local infection or poor soft-tissue condition; (4) presence of chronic ankle instability or Achilles tendon contracture; (5) scoliosis, hip dysplasia/dislocation or genu varum/valgum; (6) other lower limb deformities; history of ankle–foot injury or chronic overuse injuries (e.g., heel pain, plantar fasciitis, and plantar plate inflammation) within the past 3 months; and (7) inability to complete the study due to other factors.

### Methods

#### Treatment Methods

##### Perioperative Management and Pre-operative Preparation

All surgeries were performed by the same team of senior orthopaedic foot and ankle specialists.

1. Perioperative Management: All patients underwent routine blood tests, lower extremity arteriovenous colour Doppler ultrasound, weight-bearing anteroposterior, oblique and lateral foot X-rays, CT scans and MRI of the affected side. Prophylactic intravenous antibiotics were administered once preoperatively and within 24 h postoperatively.
2. Pre-operative Preparation: Combined spinal–epidural anaesthesia was administered with the patient positioned supine. A tourniquet was applied to the middle-upper 1/3 of the ipsilateral thigh, followed by routine disinfection with iodine tincture and alcohol and application of sterile drapes. Tourniquet pressure was set to 45 kPa.

##### ESWT Combined with Dual Stabilisation Group (Intervention Group, IG)

**Fig. 1:**
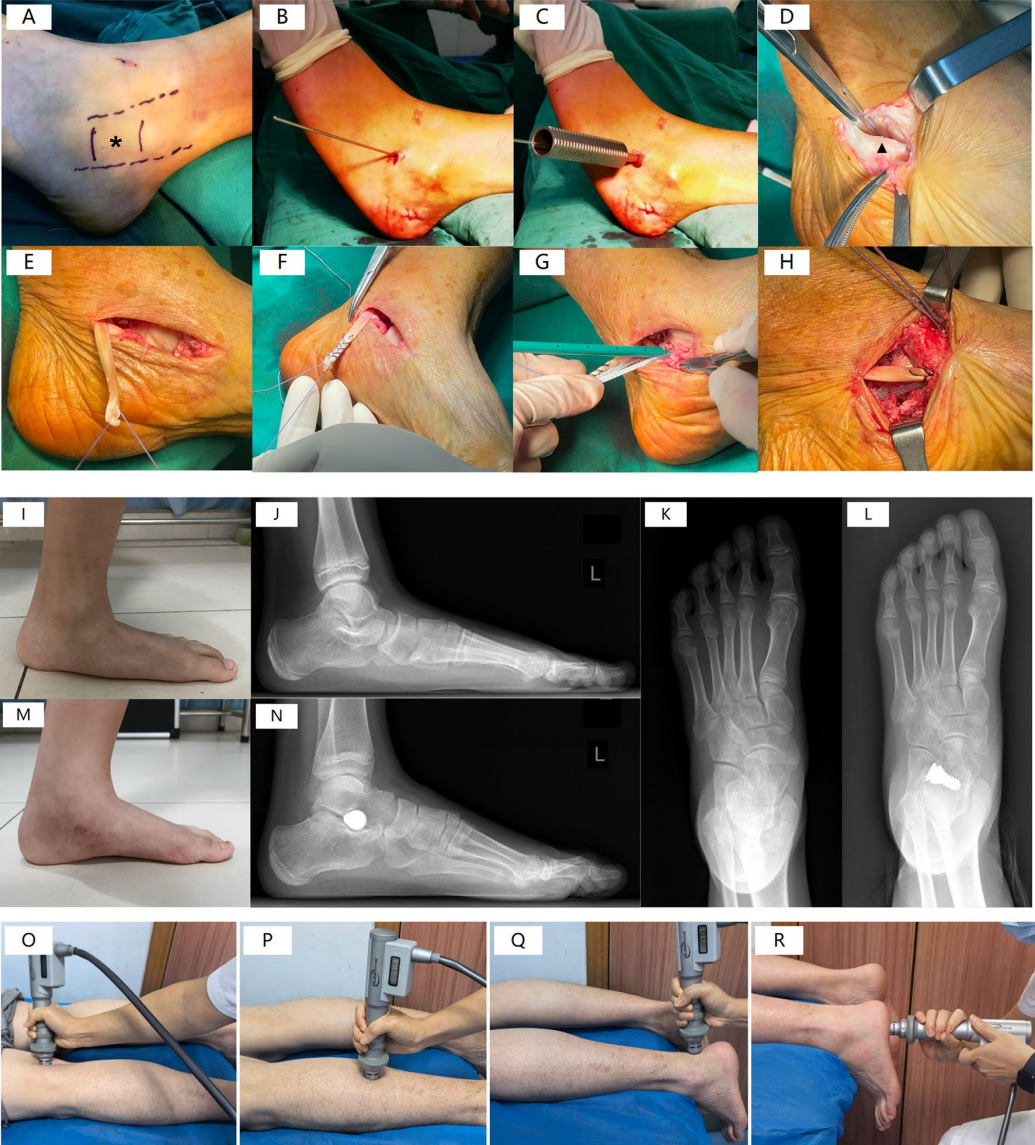
A 15-year-old male patient with PCFD Ⅰ ABCD (left foot) underwent ESWT combined with HyProCure and medial column stabilisation (DSP). **A** Schematic diagram of the incision for subtalar arthroereisis (* indicates the needle insertion point); **B**–**C** procedure for HyProCure implantation; **D** ▲ indicates hypertrophy and degeneration of the tibialis posterior tendon; **E**–**H** augmentation repair of the tibialis posterior tendon and spring ligament complex to enhance stability of the medial column; **I** preoperative appearance showing collapse of the medial arch under weight-bearing; **J**, **K** preoperative X-ray reveals collapse of the medial arch, with a T1–MT of 20°, TNCA of 22°, Meary’s of 18°, and Pitch of 16°; **M** 1-year postoperative appearance shows significant improvement in the medial arch under weight-bearing; **L**, **N** 1-year postoperative X-ray shows restoration of the medial arch, with a T1–MT of 7°, TNCA of 6°, Meary’s of 4°, and Pitch of 25°; **O**–**R** schematic diagram of extracorporeal shock wave therapy procedure

- 1) Pre-operative ESWT Pre-intervention Stage
  Patients received ESWT once weekly, scheduled 2 weeks preoperatively and from weeks 3 to 8 postoperatively, totalling eight sessions as one treatment course. The specific procedure was as follows.
  An extracorporeal shock wave device (Model O2B, Shenzhen Dejarsystem Technology Co., Ltd., China) was utilised. Prior to treatment, the patients were fully informed of the treatment process and precautions. The patient was placed in the prone position, with coupling gel applied to the skin over the ventral aspect of the affected calf and ankle. The operator held the device handle with one hand whilst stabilising the patient’s ankle with the other. The shock wave frequency was set to 5–10 Hz, treatment pressure to 2.5–4 bar and the number of shocks per session to 2000. The shock wave probe was first placed on the skin surface over the gastrocnemius muscle; with pressure applied to the handle, the probe was moved sequentially from the proximal gastrocnemius below the knee → Achilles tendon → plantar fascia, delivering 1000 shocks with medium-to-high hand pressure. Initially, lower-pressure shock waves were employed, with parameters gradually adjusted based on patient tolerance (Fig. 1O-R).
- 2) Operative Technique
  Intraoperatively, subtalar arthroereisis was performed first. A 1.5 cm incision was made in the sinus tarsi region, 1.5 cm anterior to the lateral malleolus of the affected side. The skin was sequentially incised, and blunt dissection was conducted with haemostats to separate subcutaneous tissues layer by layer, ensuring protection of surrounding structures and maintaining a clear surgical field. Then, the sinus tarsi was gradually exposed, followed by excision of a portion of the interosseous talocalcaneal ligament using tissue scissors. A guide pin was inserted into the tarsal canal, and trial implants (typically sizes 5–7) were used until arch correction was achieved with no significant limitation of dorsiflexion. An appropriately sized subtalar joint stabiliser was selected for the patient; its position was evaluated by combined fluoroscopy and visual inspection, with the degree of correction determined using the ‘metatarsal-navicular visibility sign’. C-arm fluoroscopy confirmed satisfactory positioning of the trial implant within the tarsal sinus canal, with adequate arch restoration and hindfoot valgus alignment correction. If necessary, concomitant correction of forefoot supination–abduction and adjustment of the relative relationship between the first and fifth metatarsal heads were performed. Then, the appropriately sized subtalar joint stabiliser was inserted, and re-fluoroscopy confirmed that the subtalar stabiliser was located within the tarsal canal, with satisfactory correction of the Meary’s and pitch angles.
  Subsequently, medial column stabilisation was performed. A 4 cm incision was made on the medial aspect of the affected foot, extending from the medial malleolus to the medial navicular tuberosity. The skin and subcutaneous tissues were sequentially incised, and the navicular tuberosity was gradually exposed. If preoperative radiographs had identified an accessory navicular and intraoperative exploration confirmed this, the accessory navicular was excised under direct vision whilst preserving as much of the PTT insertion as possible. This step was omitted if no accessory navicular was present. Then, peripheral inflammatory tissue was debrided. If PTT exhibited elongation, degeneration or tearing, it was resected, and the residual PTT was woven with high-strength suture in a non-locking pattern for subsequent use. After fully exposing the PTT insertion, blunt dissection was performed to reveal the spring ligament (SL). If the SL was intact, the plantar–medial surface of the navicular was freshened, and a 3.0 mm absorbable anchor (Arthrex, Naples, FL, USA) was fixated to the plantar–medial navicular; the anchor tail suture was used for simple plication of the PTT or reconstruction of its insertion. If the SL was only lax, the same technique was applied to plicate the SL using absorbable anchors. For severely torn or degenerated SL, reconstruction was performed using the InternalBrace absorbable knotless suture tape (Arthrex, Naples, FL, USA), following this procedure: a suture-reinforced composite absorbable knotless anchor was implanted into the calcaneal sustentaculum tali; the suture tail of the woven residual PTT and high-strength suture tape from the original anchor were jointly passed through another absorbable knotless suture anchor, which was then screwed into the plantar-medial navicular after adjusting suture tape tension (with the ankle maintained in neutral position, arch elevated, and foot inverted). Fluoroscopy confirmed satisfactory positioning, with restoration of Meary’s and pitch angles under simulated weight-bearing. The wound was irrigated, and after verifying stability, 2–0 absorbable sutures were used for imbricated closure of the joint capsule and deep fascia to enhance stability. Following confirmation of correct instrument and gauze counts, the skin was sutured, sterile dressings were applied, and appropriate elastic bandaging was performed. Postoperatively, short-leg plaster immobilisation was applied (Fig. 1A-N).
- 3) Postoperative ESWT Rehabilitation Phase
  ESWT rehabilitation was initiated in postoperative week 3, administered once weekly for 6 weeks, with the specific procedure consistent with that described above. During the intervention, attention was paid to wound healing, and excessive manipulation was avoided to prevent adverse effects (Fig. 1O-R).

##### Dual Stabilisation Group (Control Group, CG)

The operative technique is the same as described above.

##### Standardised Postoperative Rehabilitation Guidance of Both Groups

1. Isometric contraction training of major muscle groups was initiated on postoperative day 2.
2. After cast removal at 2 weeks postoperatively, partial weight-bearing training was implemented with an ankle–foot orthosis instead.
3. At 4 weeks postoperatively, under the supervision of rehabilitation specialists, active and passive range-of-motion exercises for the ankle (e.g., plantar flexion, dorsiflexion, inversion, and eversion) and muscle strength enhancement training were initiated.
4. For patients with smooth rehabilitation progress, endurance and proprioceptive balance training were gradually introduced between 6 and 8 weeks postoperatively, with a gradual transition to full weight-bearing ambulation.

All patients were treated with standardised preventive protocols and postoperative rehabilitation safeguards. Evaluations were conducted at 2 months, 6 months postoperatively, and during the final follow-up, utilising weight-bearing anteroposterior, oblique and lateral foot X-rays; hindfoot alignment views (the Saltzman method); foot computed tomography (CT) scans; and magnetic resonance imaging (MRI), respectively.

### Postoperative Measurement and Clinical Evaluation

Patients’ demographic data were documented. Comparisons were made between groups regarding imaging parameters, American Orthopaedic Foot and Ankle Society (AOFAS) ankle–hindfoot scores [13], Visual Analogue Scale (VAS) scores [14], Tegner activity scores [15] and time to return to athletic activity [16]. The postoperative complications in each group were also tallied.

1. Weight-bearing X-ray measurement parameters included T1-MT, TNCA, Pitch angle, Meary’s angle and CVA.
2. Elastic modulus: Young’s modulus values (kPa) measured by real-time shear wave elastography were recorded. Gastrocnemius measurement site: The lower segment of the junction between the two gastrocnemius bellies (the region of maximum muscle stiffness) was selected as the target detection point. Achilles tendon measurement site: The mid-segment of the Achilles tendon (2 cm proximal to its calcaneal insertion). The probe was positioned over the muscle belly, ensuring a minimum distance of 5 mm from bone and fascial surfaces. Each measurement was repeated three times, with the mean value recorded. All procedures were performed by senior physicians with over a decade of ultrasound experience.
3. AOFAS score: The scale includes three dimensions, namely pain, function and alignment, with a total score of 100 points. A higher score indicates better foot function.
4. VAS score ranges from 0 to 10 points, with a score of 0 indicating ‘no pain’ and 10 indicating ‘unbearable severe pain’. Patients self-rated their pain based on subjective experience.
5. Tegner activity score: Ranging from 0 to 10 points, this system assesses individual athletic ability by stratifying it into 11 grades. Each grade corresponds to distinct activity levels and physical demands, with higher scores reflecting greater athletic ability of the participant.

## Results

### Comparison of Baseline Data

Comparisons of baseline data between the two groups, including sex, age, body mass index, PCFD Stage I A/B/C/D classification, disease duration and follow-up duration, revealed no significant differences (*P* > 0.05), confirming comparability. Follow-up duration was a minimum of 12 months, with a mean of (13.66 ± 1.65) months. No significant difference was observed in hospital stay between the two groups (*P* > 0.05) (Table 1).

**Table 1:** Comparison of baseline data between the two groups

Item	IG (*n* = 36)	CG (*n* = 32)	*P* value
Mean age (years)	19.56 ± 12.14	20.66 ± 13.73	0.727
Male/female	23/13	20-Dec	0.906
Right/left	21/15	18/14	0.862
Mean BMI (kg/m2)	19.61 ± 1.38	19.38 ± 1.33	0.488
PCFD Ⅰ (A/B/C/D)			
1C	3	3	0.679
1AB	5	4	
1AC	6	4	
1BC	7	6	
1CD	4	4	
1ABC	3	3	
1ABD	2	3	
1BCD	1	1	
1ABCD	5	4	
Mean time from initial injury to surgery (weeks)	13.97 ± 3.65	14.38 ± 4.06	0.668
Mean duration of follow-up (months)	13.50 ± 1.73	13.84 ± 1.55	0.394
Hospital stay (days)	7.78 ± 1.27	7.84 ± 1.46	0.843

### Comparison of Foot Function

**Fig. 2:**
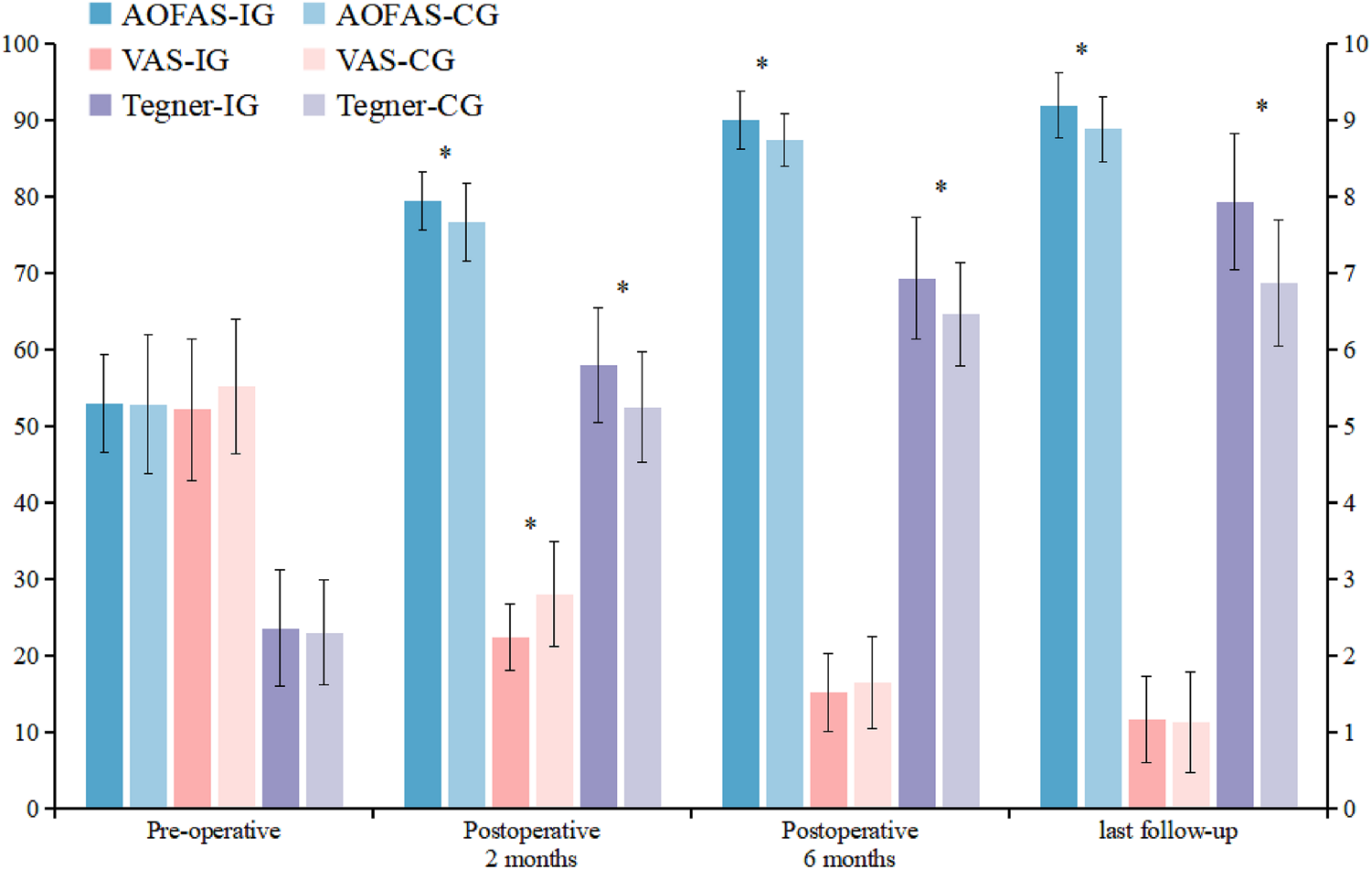
Graphical representation of the change in AOFAS scores, VAS scores and Tegner scores ($$\overline{x} \pm s$$$$\overline{x} \pm s$$), IG I Group, CG C Group. *P value < 0.05

Both groups exhibited higher AOFAS and Tegner activity scores postoperatively compared with their preoperative values within the same group, whereas their VAS scores were lower relative to their preoperative measurements. Notably, the intervention group showed higher AOFAS and Tegner activity scores than the control group at 2 and 6 months postoperatively and at the last follow-up (*P* < 0.05) and a lower VAS score at 2 months postoperatively (*P* < 0.05) (Table 2 and Fig. 2).

**Table 2:** Comparison of AOFAS scores, VAS scores and Tegner scores at different time points between the two groups

Item	Pre-operative	Postoperative 2 months	Postoperative 6 months	Last follow-up	*F*	*P* value
AOFAS						
IG (*n* = 36)	53.03 ± 6.33	79.56 ± 3.84	90.11 ± 3.77	91.97 ± 4.23	277.55	< 0.001
CG (*n* = 32)	52.91 ± 9.09	76.75 ± 5.09	87.53 ± 3.44	88.89 ± 4.30	163.21	< 0.001
* t*	0.065	2.583	2.936	2.992		
* P* value	0.949	0.012	0.005	0.004		
VAS						
IG (*n* = 36)	5.22 ± 0.93	2.25 ± 0.44	1.53 ± 0.51	1.17 ± 0.56	254.24	< 0.001
CG (*n* = 32)	5.53 ± 0.88	2.81 ± 0.69	1.66 ± 0.60	1.13 ± 0.66	238.23	< 0.001
* t*	−1.404	−4.045	−0.956	−0.281		
* P* value	0.165	< 0.01	0.343	0.779		
Tegner						
IG(*n* = 36)	2.36 ± 0.76	5.81 ± 0.75	6.94 ± 0.79	7.94 ± 0.89	327.55	< 0.001
CG (*n* = 32)	2.31 ± 0.69	5.25 ± 0.72	6.47 ± 0.67	6.88 ± 0.83	323.02	< 0.001
* t*	0.274	3.112	2.656	5.088		
* P* value	0.785	0.003	0.009	< 0.01

### Comparison of T1-MT, TNCA, Pitch Angle, Meary’s Angle, CVA, Elastic Modulus of the Achilles Tendon/Gastrocnemius (kPa) and Time to Return to Athletic Activity

**Fig. 3:**
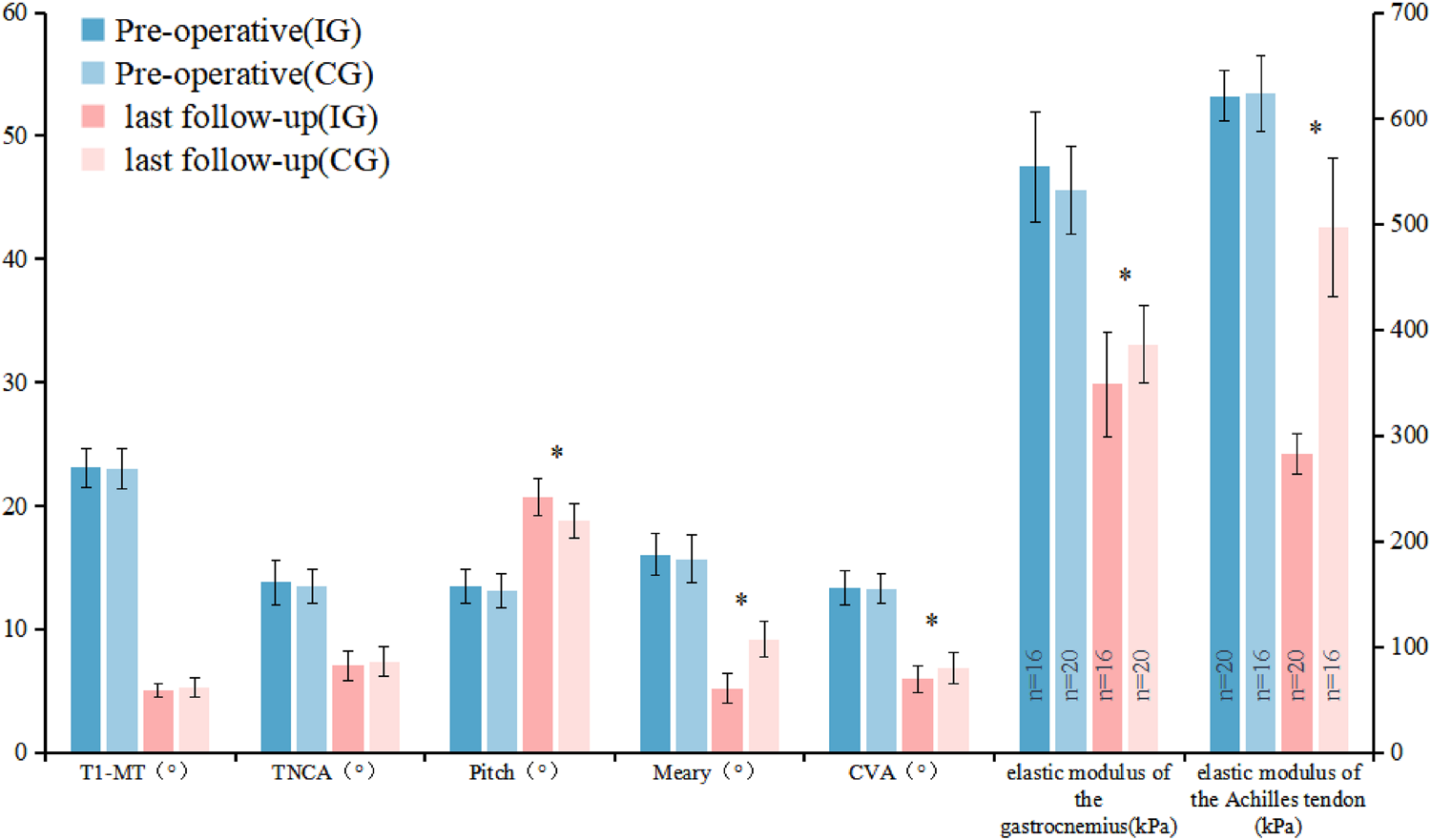
Graphical representation of the change in T1-MT, TNCA, Pitch Angle, Meary’s Angle, CVA, Elastic Modulus of the Achilles Tendon/Gastrocnemius($\overline{x} \pm s$), IG I Group, CG C Group. *P value < 0.05

Both groups demonstrated significant improvements in weight-bearing X-ray parameters (T1-MT, TNCA, pitch angle, Meary’s angle, CVA) and elastic modulus of the Achilles tendon/gastrocnemius postoperatively compared with preoperative measurements (*P* < 0.05). At final follow-up, the intervention group showed superior outcomes in CVA and elastic modulus of the Achilles tendon/gastrocnemius relative to the control group (*P* < 0.05), although no significant intergroup differences were noted in anteroposterior T1-MT, TNCA, pitch angle or Meary’s angle at final follow-up (*P* > 0.05). During follow-up, 59 patients (86.76%) in both groups met the criteria for returning to athletic activity. The intervention group achieved a shorter time to return to athletic activity [(6.26 ± 1.26) months; 33 cases, 91.67%] compared with the control group [(8.24 ± 2.06) months; 26 cases, 81.25%], with a significant difference (*P* < 0.05) (Table 3 and Fig. 3).

**Table 3:** Comparison of T1-MT, TNCA, Pitch Angle, Meary’s Angle, CVA, Elastic Modulus of the Achilles Tendon/Gastrocnemius (kPa) and return to sports time between the two groups

Item		IG(*n* = 36)	CG(*n* = 32)	*t*	*P* value
T1-MT (°)					
Pre-operative	23.10 ± 1.61	23.03 ± 1.59	0.186	0.853	Pre-operative
Last follow-up	5.04 ± 0.57	5.29 ± 0.74	−1.562	0.123	Last follow-up
* t*	63.034	65.243			*t*
* P* value	< 0.001	< 0.001			*P* value
TNCA (°)					
Pre-operative	13.86 ± 1.81	13.47 ± 1.37	1.000	0.321	Pre-operative
Last follow-up	7.06 ± 1.24	7.38 ± 1.19	0.283	18.407	Last follow-up
* t*	24.964	−1.082			*t*
* P* value	< 0.001	< 0.001			*P* value
Pitch (°)					
Pre-operative	13.11 ± 1.37	13.50 ± 1.44	−1.142	0.257	Pre-operative
Last follow-up	20.36 ± 1.31	20.78 ± 1.50	−1.233	0.222	Last follow-up
* t*	−23.041	−18.452			*t*
* P* value	< 0.001	< 0.001			*P* value
Meary (°)					
Pre-operative	15.72 ± 1.89	16.09 ± 1.71	−0.845	0.401	Pre-operative
Last follow-up	6.36 ± 1.64	5.81 ± 1.15	1.578	0.119	Last follow-up
* t*	26.410	25.723			*t*
* P* value	< 0.001	< 0.001			*P* value
CVA (°)					
Pre-operative	13.39 ± 1.34	13.31 ± 1.23	0.244	0.808	Pre-operative
Last follow-up	5.97 ± 1.08	6.91 ± 1.25	−3.298	0.002	Last follow-up
* t*	26.228	21.543			*t*
* P* value	< 0.001	< 0.001			*P* value
Elastic modulus of the gastrocnemius (kPa)					
Pre-operative	620.93 ± 23.83(*n* = 20)	623.24 ± 35.94(*n* = 16)	−0.215	0.831	Pre-operative
Last follow-up	282.69 ± 19.16(*n* = 20)	497.38 ± 65.49(*n* = 16)	−13.793	< 0.001	Last follow-up
* t*	45.868	6.143			*t*
* P* value	< 0.001	< 0.001			*P* value
Return to sports time (months)		6.26 ± 1.26	8.24 ± 2.06	−4.671	< 0.001

### Postoperative Complications

In the intervention group, one case developed recurrence of flatfoot deformity after subtalar joint stabiliser removal; symptoms improved following professional rehabilitation guidance and use of orthotic insoles. In the control group, one case presented with poor healing of a medial foot wound secondary to infection, which resolved after anti-infective therapy and wound debridement. During follow-up, the subtalar joint stabilisers and anchors in both groups demonstrated good biocompatibility, with no other adverse events observed.

## Discussion

With increasing living standards in modern society, the incidence of flatfoot is increasing alongside growing rates of overweight and obesity. Flexible flatfoot is characterised by medial arch collapse and hindfoot valgus under weight-bearing, arising from alterations in dynamic and static stabilising structures. It presents with foot pain or fatigue during prolonged standing or walking. Flexible flatfoot is relatively prevalent in China, with reported incidences ranging from 0.8 to 3.7%, whereas international data indicate a higher range of 2.7–16.4% [17, 18]. Without standardised management, it may progress to rigid flatfoot, potentially inducing compensatory internal rotation of the tibia and femur and ultimately leading to significant lower limb alignment deviations [19]. Thus, timely and appropriate intervention can prevent further deterioration of flatfoot deformity. Conservative treatments, including arch supports, stretching exercises and orthoses, are associated with long treatment cycles and high compliance requirements, yielding suboptimal outcomes in patients with severe flatfoot [20]. For those unresponsive to conservative therapy for 3–6 months, surgical intervention may be considered. Surgical options such as osteotomy, lateral column lengthening, arthrodesis and tendon transfer are associated with substantial foot trauma, risks of delayed or non-union of osteotomies, prolonged recovery of foot biomechanics and potential impacts on normal skeletal development. Furthermore, these procedures cause significant alterations to native anatomical structures; in cases of surgical failure, subsequent salvage measures are highly limited, resulting in diminished quality of life for patients.

‘PCFD’ was proposed by nine international foot and ankle experts following a series of discussions and votes, with the final consensus statement published in 2020 in the *Foot & Ankle International* journal. The consensus group opted for this new term in lieu of ‘PTT dysfunction’ or ‘adult-acquired flatfoot deformity’ for several compelling reasons: First, this deformity is not rooted in the linear progression of PTT; PTT is merely one of the multiple structures involved in this complex pathological process. Second, the medial ligament complex, particularly the SL and deltoid ligament, and deformities involving the tarsometatarsal or cuneonavicular joints in flatfoot may require correction of foot instability beyond PTT. Third, the term ‘progressive’ indicates that the disease may deteriorate over time, accompanied by other deformities. Fourth, ‘collapsing foot deformity’ emphasises its pathological and progressive nature, distinguishing it from ordinary flatfoot.

Subtalar joint stability is critical in PCFD. The subtalar joint is a weight-bearing joint that bears and transmits body weight, converts rotational forces of the lower limb, coordinates and assists movements of lower limb joints and governs midtarsal and forefoot activities through the transmission and distribution of body weight [21]. Peritalar joint movements depend on the subtalar joint; in PCFD, talar head depression, interosseous ligament laxity and loss of normal biomechanical structure are common, leading to articular cartilage wear, foot instability and pain. Without disrupting osseous structures, subtalar arthroereisis is minimally invasive, corrects calcaneal valgus and abnormal talar adduction/plantar flexion, preserves proprioception, restores foot biomechanics and normal development and is well tolerated by patients, thus preventing further progression of flatfoot [22]. In the present study, intraoperative fluoroscopy revealed that after HyProCure implantation into the sinus tarsi, simulated weight-bearing anteroposterior T1-MT, TNCA, pitch angle, Meary’s angle and CVA improved to varying degrees. This indicates that HyProCure can enhance and stabilise the talotarsal joint complex and medial longitudinal arch, prevent excessive arch collapse, restore arch height and correct hindfoot valgus.

Clinically, patients with flexible flatfoot are often accompanied by Achilles tendon/gastrocnemius contracture. Studies determined that calf muscle contracture is a key factor contributing to tension in the posterior calf and plantar muscles, which exacerbates arch collapse and hindfoot valgus [23, 24]. Reports and clinical observations have indicated that PCFD is frequently associated with Achilles tendon/gastrocnemius contracture [25]. In PCFD treatment, combined osteotomy, subtalar arthroereisis or soft-tissue plication can correct deformities and restore lower limb alignment; however, open release of the gastrocnemius aponeurosis or percutaneous Achilles tendon release may lead to surgical complications (most commonly sural nerve injury) and increase patient discomfort [26]. ESWT has shown favourable efficacy in treating myofascial pain syndrome by achieving mechanical release with varying pressure intensities by physical manipulation, promoting microcirculation. It also yields good results in managing Achilles tendon contracture, gastrocnemius injury and fasciitis [27]. Studies have revealed that Achilles tendinopathy and gastrocnemius contracture are highly similar. The Achilles tendon, formed by the conjoined distal extension of the medial and lateral gastrocnemius heads and the soleus muscle, exhibits intricate interconnections and mutual influences. Thus, ESWT is beneficial for addressing Achilles tendon and gastrocnemius contracture [28]. This may be attributed to ESWT stimulating fibroblasts in muscles to secrete mitogens, promoting intracellular organelle and cytoplasmic movement, enhancing metabolism, improving tissue oxygenation and facilitating repair. In this study, the intervention group received ESWT combined with HyProCure and medial column stabilisation, which released the mechanical hinge mechanism in PCFD, restored lower limb alignment and flexible tensile forces of the foot, corrected rotational deformities of peritalar joints to enhance stability and reduced complications associated with open surgery. This approach corrected weight-bearing radiographic parameters (CVA) and improved the elastic modulus of the gastrocnemius and Achilles tendon, thereby restoring muscle and tendon function. This finding suggests that, in addition to correcting arch collapse and restoring hindfoot alignment, adjunctive ESWT effectively modulates the biomechanical properties of the posterior calf soft tissues. The further reduction of CVA not only indicates a more complete correction of hindfoot valgus deformity but also reflects optimization of weight-bearing transmission and support points. Meanwhile, the decrease in elastic modulus highlights enhanced compliance and reduced contracture of the gastrocnemius–Achilles complex, thereby alleviating localised tendon stress and improving tolerance to dynamic loading. Compared with traditional open lengthening procedures, ESWT provides a minimally invasive, repeatable, and safe modality that reduces the risk of surgical complications whilst promoting tendon repair and metabolic recovery. These advantages are consistent with recent perspectives emphasising the restoration of “dynamic stability” in the management of PCFD, thereby offering additional evidence in support of combined surgical and non-invasive interventions.

In PCFD treatment, medial column stability is equally crucial, serving as a key mechanical hinge between the forefoot and hindfoot, particularly regarding PTT and deltoid ligament. During the development of paediatric flatfoot, accessory navicular is common, often accompanied by medial arch pain due to foot strain or increased pressure, which affects walking, athletic activity and even lower limb mechanical development. Surgical intervention should be considered if conservative treatments yield poor outcomes. In adults, acquired flatfoot often develops due to PTT insufficiency and medial column instability. In adolescents with PCFD and an accessory navicular, anatomical abnormalities associated with the accessory navicular lead to displacement of the native navicular insertions of the PTT and SL, resulting in local prominence and tenderness at the navicular tuberosity. With increased load-bearing activities, the PTT and SL gradually elongate, degenerate or tear; thus, surgery should involve PTT repair or insertion reconstruction and SL repair/reconstruction to restore medial column stability [29]. Decreased strength of the PTT or SL increases plantar pressure during weight-bearing, initiating a vicious cycle that damages foot stabilising structures, exacerbating flatfoot and even impairing ambulation. Although isolated SL injury can cause arch collapse, forefoot abduction and other flatfoot deformities, its incidence is relatively low. Therefore, some scholars argue that SL repair/reconstruction is unnecessary if the foot demonstrates adequate inversion mobility following sufficient correction [30]. In the present study, three patients required simultaneous PTT repair and SL plication, indicating a low incidence of SL injury in PCFD, which is consistent with current epidemiological data on the SL. Additionally, previous studies reported that restoring medial–lateral mechanical balance in flexible flatfoot requires more than isolated medial column stabilisation; it often warrants combined use of other stabilisation procedures (e.g., subtalar arthroereisis and lateral column lengthening) and correction of forefoot supination deformity [14].

The anatomical and functional stability of the foot is categorised into dynamic and static components. Dynamic stability relies on muscles and tendons, with PTT playing a critical role in maintaining the dynamic stability of the medial flatfoot structure. Static stability is maintained by bones and ligaments. Procedures such as medial cuneiform Cotton osteotomy, calcaneal osteotomy and lateral column lengthening disrupt osseous structures, cause significant trauma and are poorly tolerated by patients. In contrast, the SL, which is a key contributor to medial static stability, is particularly important. Biomechanical models have demonstrated that flatfoot deformities emerge with minimal loading following SL transection [31]. In this study, all PCFD cases were flexible without overt osteoarthritis. For patients with an accessory navicular, surgery involved accessory navicular resection combined with PTT insertion reconstruction or SL repair/reconstruction. This combined dynamic–static stabilisation addressed medial mechanical issues of the foot, whereas concurrent subtalar arthroereisis resolved subtalar instability and corrected forefoot supination deformity, enabling earlier weight-bearing and faster return to normal shoe wear. Postoperative follow-up showed ankle–foot function recovery in both groups; both groups exhibited higher postoperative AOFAS and Tegner activity scores than their preoperative values within the same group, whereas their VAS scores were lower relative to their preoperative measurements. Notably, the intervention group showed higher AOFAS and Tegner activity scores than the control group at 2 and 6 months postoperatively and at the last follow-up (*P* < 0.05) and a lower VAS score at 2 months postoperatively (*P* < 0.05). These confirm the significant clinical efficacy of ESWT combined with HyProCure and medial column stabilisation in treating PCFD, attributed to the following. First, PTT substantially contributes to medial column stability in PCFD, making restoration of medial column stability pivotal for flatfoot management. Second, accessory navicular is common in adolescent patients, where a painful accessory navicular and PTT degeneration coexist. Resection of the accessory navicular combined with reconstruction of the PTT insertion effectively alleviates pain and corrects dynamic mechanical abnormalities of the foot. Third, ESWT-mediated release of Achilles tendon/gastrocnemius contracture significantly improves elastic modulus, enhances function and assists in adjusting muscle mechanical balance of the foot and optimising lower limb alignment.

The current study focussed on flexible (rather than rigid) PCFD, specifically stage I. Moreover, this study aimed to restore dynamic and static stabilising structures of the collapsed foot from a biomechanical perspective using HyProCure and medial column stabilisation as primary interventions, supplemented by ESWT. After treatment, both groups showed significant improvements in weight-bearing X-ray parameters (i.e. T1-MT, TNCA, pitch angle, Meary’s angle and CVA) compared with preoperative values (*P* < 0.05). At final follow-up, the intervention group demonstrated superior outcomes in CVA and elastic modulus of the Achilles tendon/gastrocnemius (*P* < 0.05), restoring hindfoot morphology and effective weight-bearing support points. Improved elastic modulus of the Achilles tendon/gastrocnemius enhanced heel-rise capacity and plantigrade propulsion. Clinically, both groups showed increased AOFAS and Tegner activity scores and decreased VAS scores, relieving pain and restoring ankle–foot mobility. Postoperative statistics revealed 1 case of flatfoot deformity recurrence in the intervention group after subtalar stabiliser removal (1/36, 2.78%) and 1 case of poor healing of a medial foot wound due to infection in the control group (1/32, 3.13%). No other severe complications occurred in either group, indicating that DSP for PCFD are associated with low overall complication rates (2.94%) and high patient satisfaction.

Facing high-intensity work and life pressures, contemporary individuals rely on exercise—often requiring appropriate physical activity to regulate the dopaminergic system and promote physical and mental health [32]. Restoring minimal daily walking function no longer fully meets most patients’ needs; instead, their primary desire is to regain high-intensity and athletic function. During follow-up, 60 patients (88.24%) in both groups met the criteria for returning to athletic activity. The intervention group returned to athletic activity more rapidly [(6.26 ± 1.26) months; 33 cases, 91.67%] than the control group [(8.24 ± 2.06) months; 26 cases, 81.25%], showing a significant difference (*P* < 0.05). Tegner scores significantly improved in both groups compared with preoperative values (*P* < 0.05), with higher scores in the intervention group (*P* < 0.05), better meeting patients’ criteria for returning to athletic activity.

This study has several inherent limitations. First, the retrospective design introduces potential selection and information bias, as all clinical and imaging data were extracted from medical records rather than collected prospectively. Second, the absence of randomisation may limit the comparability between groups, even though baseline characteristics showed no significant differences. Third, follow-up duration averaged 13.66 months, which does not fully reflect long-term tendon remodelling, recurrence rates or implant survival. Fourth, although elastography provides objective quantification of gastrocnemius and Achilles tendon stiffness, measurements were operator-dependent and not repeated by multiple examiners, which may affect reliability. Future prospective studies with larger sample sizes, longer follow-up periods and randomised-controlled designs are warranted to validate these findings.

Therefore, ESWT combined with HyProCure and medial column stabilisation alleviates pain, improves functional outcomes and is associated with few complications in PCFD treatment, thereby facilitating earlier return to athletic activity.

## Data Availability

The datasets generated and analysed during the current study are available from the corresponding author on reasonable request.

## References

[1] Jay, R. M., & Din, N. (2013). Correcting pediatric flatfoot with subtalar arthroereisis and gastrocnemius recession: A retrospective study. *Foot & Ankle Specialist,**6*(2), 101–107. 23263679 10.1177/1938640012470714

[2] Li, B., He, W., Yu, G., et al. (2021). Treatment for flexible flatfoot in children with subtalar arthroereisis and soft tissue procedures. *Frontiers in Pediatrics,**9*, 656178. 34095026 10.3389/fped.2021.656178 PMC8175848

[3] Kothari, A. (2016). Are flexible flat feet associated with proximal joint problems in children? *Gait & Posture,**45*, 204–210. 26979907 10.1016/j.gaitpost.2016.02.008

[4] Shi, C., Li, M., Zeng, Q., et al. (2023). Subtalar arthroereisis combined with medial soft tissue reconstruction in treating pediatric flexible flatfoot with accessory navicular. *Journal of Orthopaedic Surgery and Research,**18*(1), 55. 36658597 10.1186/s13018-023-03542-w PMC9850534

[5] Cai, B., Xiao, K., & Xie, M. (2023). Spring ligament reefing with talonavicular fixation for correction of adult flatfoot. *Orthopedic Journal of China,**31*(21), 2005–2008.

[6] Liu, F., Zhang, Z. M., Zhang, C., et al. (2024). Effect observation of distal joint immobilization combined with soft tissue balance and osteotomy on the treatment of adolescent flat foot combined with hallux valgus deformity. *Chinese Journal of Bone Joint Injury,**39*(10), 1112–1115.

[7] Liang, F., Wang, Z., Wei, F. Y., et al. (2017). Treatment of gastrocnemius contracture. *Orthopedic Journal of China,**25*(7), 632–634.

[8] Stania, M., Juras, G., Chmielewska, D., et al. (2019). Extracorporeal shock wave therapy for achilles tendinopathy. *BioMed Research International,**2019*, 1–13. 10.1155/2019/3086910 PMC6948318 31950037

[9] Lee, H. J., Lim, K. B., Yoo, J., et al. (2015). Effect of custom-molded foot orthoses on foot pain and balance in children with symptomatic flexible flat feet. *Annals of Rehabilitation Medicine,**39*(6), 905–913. 26798604 10.5535/arm.2015.39.6.905 PMC4720766

[10] Taha, A. M. S. (2015). Painful flexible flatfoot. *Foot and Ankle Clinics,**20*(4), 693–704. 26589087 10.1016/j.fcl.2015.07.011

[11] Myerson, M. S., Thordarson, D., Johnson, J. E., et al. (2020). Classification and nomenclature: Progressive collapsing foot deformity. *Foot and Ankle International,**41*(10), 1271–1276. 32856474 10.1177/1071100720950722

[12] Duthon, V. B., Lubbeke, A., Duc, S. R., et al. (2011). Noninsertional achilles tendinopathy treated with gastrocnemius lengthening. *Foot & Ankle International,**32*(4), 375–379. 21733439 10.3113/FAI.2011.0375

[13] Yan, G. B. (2014). AOFAS ankle hindfoot scale. *Chinese Journal of Joint Surgery (Electronic Edition),**8*(4), 557.

[14] Gross, C. E., & Jackson, J. B. (2021). The importance of the medial column in progressive collapsing foot deformity. *Foot and Ankle Clinics,**26*(3), 507–521. 34332732 10.1016/j.fcl.2021.06.001

[15] Qureshi, A. R., & Kay, J. (2023). Anatomical double-bundle anterior cruciate ligament reconstruction moderately improved Tegner scores over the long-term: A systematic review and meta-analysis of randomized controlled trials. *Knee Surgery, Sports Traumatology, Arthroscopy,**64*(5), 436–448. 10.1007/s00167-022-07046-8 35838793

[16] D’Hooghe, P., Cruz, F., & Alkhelaifi, K. (2020). Return to play after a lateral ligament ankle sprain. *Current Reviews in Musculoskeletal Medicine,**13*(3), 281–288. 32377961 10.1007/s12178-020-09631-1 PMC7251008

[17] Zhang, J., Jiang, S. Y., Li, Y., et al. (2023). Advances in diagnosis, prevention and treatment of flexible flatfoot in children. *Chinese Journal of School Health,**44*(6), 946–950.

[18] Brijwasi, T. (2023). A comprehensive exercise program improves foot alignment in people with flexible flat foot: A randomised trial. *Journal of Physiotherapy,**69*(1), 42–46. 36526555 10.1016/j.jphys.2022.11.011

[19] Prachgosin, T., Chong, D. Y. R., Leelasamran, W., et al. (2015). Medial longitudinal arch biomechanics evaluation during gait in subjects with flexible flatfoot. *Acta of Bioengineering and Biomechanics,**17*(4), 121–130. 26898763

[20] Elsayed, W., Alotaibi, S., Shaheen, A., et al. (2023). The combined effect of short foot exercises and orthosis in symptomatic flexible flatfoot: A randomized controlled trial. *European Journal of Physical and Rehabilitation Medicine,**59*(3), 396–405. 36988565 10.23736/S1973-9087.23.07846-2 PMC10272929

[21] Ghali, A., Mhapankar, A., Momtaz, D., et al. (2022). Arthroereisis: Treatment of pes planus. *Cureus,**14*(1), e21003. 35154977 10.7759/cureus.21003 PMC8818258

[22] Bernasconi, A., Lintz, F., & Sadile, F. (2017). The role of arthroereisis of the subtalar joint for flatfoot in children and adults. *EFORT Open Reviews,**2*(11), 438–446. 29218229 10.1302/2058-5241.2.170009 PMC5706055

[23] Wang, E. B., Liu, X. T., & Yan, W. (2023). Flexible flatfoot in children and adolescents: Where are we? *Chinese Journal of Bone and Joint,**12*(9), 641–645.

[24] Gamba, C., Álvarez Gomez, C., Martínez Zaragoza, J., et al. (2022). Proximal medial gastrocnemius release: Surgical technique. *JBJS Essential Surgical Techniques,**12*(1), e20. 10.2106/JBJS.ST.20.00039 PMC9173562 35692721

[25] Smith, J. T., Michalski, M. P., Greene, B. D., et al. (2023). Isolated gastrocnemius recession for progressive collapsing foot deformity. *Journal of the American Academy of Orthopaedic Surgeons,**31*(1), 49–56. 36548153 10.5435/JAAOS-D-22-00343

[26] Digiovanni, C. W., Kuo, R., Tejwani, N., et al. (2002). Isolated gastrocnemius tightness. *The Journal of Bone and Joint Surgery-American Volume,**84*(6), 962–970. 12063330 10.2106/00004623-200206000-00010

[27] Ismail Hassan, M., Shafiek Mustafa Saleh, M., Hesham Sallam, M., et al. (2025). Extracorporeal shock wave therapy versus laser therapy in treating musculoskeletal disorders: A systematic review and meta-analysis. *Lasers in Medical Science*. 10.1007/s10103-025-04392-0 40232318 10.1007/s10103-025-04392-0 PMC12000203

[28] Zhang, C., Zhao, X., Cao, J., et al. (2021). Triple hemisection percutaneous Achilles tendon lengthening for severe ankle joint deformity. *Orthopaedic Surgery,**13*(8), 2373–2381. 34806335 10.1111/os.13096 PMC8654661

[29] Brodsky, J. W., Baum, B. S., Pollo, F. E., et al. (2005). Surgical reconstruction of posterior tibial tendon tear in adolescents: Report of two cases and review of the literature. *Foot & Ankle International,**26*(3), 218–223. 15766424 10.1177/107110070502600306

[30] Orr, J. D., & N, I. I. J. A. (2013). Isolated spring ligament failure as a cause of adult-acquired flatfoot deformity. *Foot & Ankle International,**34*(6), 818–823. 23564421 10.1177/1071100713483099

[31] Liu, F. S. H., Xu, J., Zhao, B. C., et al. (2020). Three-dimensional finite element biomechanical analysis of stage II adult acquired flatfoot deformity after medial column stabilization. *Chinese Journal of Tissue Engineering Research,**24*(18), 2805–2810.

[32] Jia, Y., Sai, X., & Zhang, E. (2024). Comparing the efficacy of exercise therapy on adult flexible flatfoot individuals through a network meta-analysis of randomized controlled trials. *Scientific Reports,**14*(1), 2118. 39261538 10.1038/s41598-024-72149-w PMC11390964

